# Changes and Relationships of Climatic and Hydrological Droughts in the Jialing River Basin, China

**DOI:** 10.1371/journal.pone.0141648

**Published:** 2015-11-06

**Authors:** Xiaofan Zeng, Na Zhao, Huaiwei Sun, Lei Ye, Jianqing Zhai

**Affiliations:** 1 State Key Laboratory of Simulation and Regulation of Water Cycle in River Basin, Institute of Water Resources of Hydropower Research, Beijing, China; 2 School of Hydropower & Information Engineering, Huazhong University of Science and Technology, Wuhan, China; 3 National Climate Centre, China Meteorological Administration, Nr. 46, Zhongguancun South Street, Haidian District, Beijing, China; University of Aveiro, PORTUGAL

## Abstract

The comprehensive assessment of climatic and hydrological droughts in terms of their temporal and spatial evolutions is very important for water resources management and social development in the basin scale. To study the spatial and temporal changes of climatic and hydrological droughts and the relationships between them, the SPEI and SDI are adopted to assess the changes and the correlations of climatic and hydrological droughts by selecting the Jialing River basin, China as the research area. The SPEI and SDI at different time scales are assessed both at the entire Jialing River basin and at the regional levels of the three sub basins. The results show that the SPEI and SDI are very suitable for assessing the changes and relationships of climatic and hydrological droughts in large basins. Based on the assessment, for the Jialing River basin, climatic and hydrological droughts have the increasing tendency during recent several decades, and the increasing trend of climatic droughts is significant or extremely significant in the western and northern basin, while hydrological drought has a less significant increasing trend. Additionally, climatic and hydrological droughts tend to increase in the next few years. The results also show that on short time scales, climatic droughts have one or two months lag impact on hydrological droughts in the north-west area of the basin, and have one month lag impact in south-east area of the basin. The assessment of climatic and hydrological droughts based on the SPEI and SDI could be very useful for water resources management and climate change adaptation at large basin scale.

## Introduction

Climate change has been recognized as one of the major threats in the twenty-first century. The Intergovernmental Panel on Climate Change (IPCC) concluded that climate has changed over the past century, which human activities have had an influence on these changes, and that climate is expected to continue to change in the future [1–2]. Even under conservative scenarios, future climate changes are likely to include further increases in mean temperature (about 2–4°C globally) with significant drying in some regions [3–4], as well as increases in frequency and severity of extreme droughts, hot extremes, and heat waves [1, 5]. The major threat of climate change is not the change in the average temperature but the increase of extreme events.

Among the extreme meteorological events, droughts are possibly the most slowly developing ones, that often have the longest duration, and at the moment the least predictable among all atmospheric hazards [6]. Drought is a recurring extreme climate event characterized by a significant deficiency of precipitation during a period of months to years and over a large area. It occurs in virtually all parts of the World thus affecting economic activities, human lives and various elements of the environment [7]. In contrast to aridity, which is a permanent feature of climate and is restricted to low rainfall areas [8], a drought is a temporary aberration to its local normal condition. Potential changes in the characteristics of drought would have adverse effects on water resources management and aquatic ecosystems [9].

As is very difficult to quantify the characteristics of drought in terms of intensity, duration, and spatial coverage, much effort has been devoted to developing techniques for monitoring drought conditions [10]. Among these, drought indices are the most widely used, because they provide a quantitative method for determining the onset and end of a drought event and indicating the level of drought severity. Depending on the form of water and its related variable of interest, drought is conventionally characterized as meteorological, hydrological or agricultural [11].

The major existing indices for characterizing a meteorological drought are either the Palmer Drought Severity Index (PDSI) [12] based on a soil water balance equation or the Standardized Precipitation Index (SPI) [13] based on a precipitation probabilistic approach. However, they both have their deficiencies. The PDSI suffers from strong influence of calibration period, problems in spatial comparability, and subjectivity in relating drought conditions to the values of the index [14]. The SPI is mainly criticized for the fact that its calculation is only related to precipitation data. The SPI does not take other variables which may influence droughts into consideration, such as temperature, evapotranspiration, wind speed, and soil water holding capacity.

In characterizing hydrological droughts, several drought indices having been developed over the years are either palmer hydrological drought index (PHDI) [12] integrating water supply with water loss in a soil moisture model or surface water supply index (SWSI) [15] incorporating snowpack, streamflow, precipitation, and reservoir storage to complement the Palmer Index for moisture conditions. The indices designed for characterizing hydrological droughts, in general, are data demanding and computationally intensive [16].

Recently, some newer drought indices have been developed to characterize meteorological droughts and hydrological droughts. The standardized precipitation evapotranspiration index (SPEI) combines the sensitivity of the PDSI to changes in evaporation demand (caused by temperature fluctuations and trends) with the simplicity of calculation and the multi-temporal nature of the SPI [14]. The SPEI is particularly suited for detecting, monitoring, and exploring the consequences of global warming on meteorological droughts. The streamflow drought index (SDI) [17], is a very simple and effective index to characterize the severity of hydrological droughts [18]. Compared to traditional drought indices, the SPEI and SDI can characterize the severity of drought more thoroughly and efficiently. The research about the relationships between the SPEI and the hydrological drought index could be seen in [19], which analyzed the response of monthly runoff to precedent climatic conditions at different temporal scales in the Ebro basin (northeast Spain).

Due to the global climate change, China has also experienced frequent drought events in recent years. Frequent severe droughts in 1997, 1999 to 2002 in many areas of northern China have caused large economic and societal losses [20]. Water shortage, desertification, and dust storms accompanied the drying climate in both rural and urban areas due to the droughts. For example, from 1972 to 1997, the Yellow River experienced drying-up (zero streamflow) episodes for 20 years, and the earlier start time and longer periods of the drying up have become more frequent since the early 1990s. As global warming, it is also reported that there has been an increased risk of droughts since the late 1970s in the Yellow River basin [21–22].

As the largest tributary of the upper Yangtze River, The Jialing River is the upstream tributary having the highest content of sediment and it is also the important flood and sediment sources for the Three Gorges project, China. The coming water of Jialing River has an important influence on the situation in water and sediment of the Three Gorges Region. In recent ten years, the runoff of Jialing River basin decreased year by year, and the floods and droughts frequently occurred alternately, especially the agricultural production was under the serious threat of droughts. Extreme drought in 1997 caused direct economic loss of about 121 million US Dollars in crop of Nanchong city which is located in the Jialing River basin. Droughts in 2006 also led to the great economic losses in Sichuan province and Chongqing city, and Chongqing city experienced the most severe drought in the history.

Under the background of global and regional climate change, to explore the comprehensive characteristics of droughts in the Jialing River basin, the newer indices including the SPEI and SDI on time scales of 3, 6, 9 and 12 months were used to assess the changes and relationships of climatic and hydrological droughts in the basin over the 1962–2010. The SPEI and SDI were firstly applied to analyze the temporal and spatial evolution of climatic and hydrological droughts for different time scales respectively. Furthermore, the results of the SPEI and SDI were used together to analyze the relationships between climatic and hydrological droughts in the whole basin and sub basins. The research focuses on the relationships between climatic drought and hydrological drought, thus to provide some early warning information for water resources department when climatic drought is happening. The possible impacts of human activities are analyzed in a simple way.

This paper is organized as follows. “Study region and data” describes the study area and data. “Methodology” introduces the SPEI and SDI, as well as the method for analyzing the changes. “Results and Discussions” shows the results and discusses the spatial and temporal changes of climatic and hydrological droughts, and the relationships between them. “Conclusions” concludes the present study.

## Study Region and Data

The Jialing River, the major tributary to the upper reaches of the Yangtze River, flows through the four provinces Shaanxi, Gansu, Sichuan and Chongqing (municipalities directly under the central government), with a mainstream length of 1120 km. The whole river drainage covers the area of 160 000 km^2^, accounting for 9% of the Yangtze River basin. The Jialing River is well-drained, which mainly involves: the Jialing River mainstream, the Qu River, the Fu River. The Qu River is the largest tributary of the left bank of the Jialing River mainstream, the total length of 665 km, accounting for 24.52% of the total area of the Jialing River basin. The Fu River is a tributary of the right bank of the Jialing River midstream, the total length of 697 km, accounting for 20.49% of the total area of the Jialing River basin.

The terrain of the whole Jialing River Basin is higher in the northwest part and is lower in the southeast part. The main Jialing River has many tributaries, which makes it like a branching river. The upstream rivers are meandering and the valleys are very deep, which often suffer slides and flash floods. The middle reaches have flat river beds and the terrain changes gradually from the deep hilly area to the shallow hilly area. The lower main river is parallel to the east of the Sichuan basin and forms to a gorge reach, and the lower basin rises to the mountainous terrain.

The Jialing River basin has seven soil types including red soil, yellow soil, brown soil, neutral purple soil, stone loess and alluvial soil. Among them, neutral purple soil, yellow soil and alluvial soil distribute widely. Due to the impacts of the topography, the distribution of vegetation in the basin is not uniform. The vegetation in the upper reaches of the river basin is relatively good, while the vegetation in the middle and lower reaches is relatively poor. The Jialing River basin is located in subtropical monsoon climate zone with the annual mean temperature of 15°C in the northern mountainous area and 17°C in the southern hilly lands. The annual mean precipitation is 935 mm, and the precipitation mainly concentrates from June to September, accounting for 66% of the annual precipitation.

In order to calculate the SPEI and SDI, monthly precipitation data, monthly average temperature data and monthly average runoff data are needed, in which monthly precipitation data and monthly average temperature data are used for calculating the SPEI, and monthly average runoff data is used for calculating the SDI.

Monthly precipitation data and monthly average temperature data from 1962 to 2010 at 16 meteorological stations are obtained from the National Climate Center of the China Meteorological Administration. The time scale of the original climate data is monthly. Among them, 2 meteorological stations (Pingwu and Fuling) only have available monthly precipitation data and monthly average temperature data from 1962 to 2008. The missing average temperature data of 1 or 2 months are replaced by the historical average.

The monthly average runoff data are collected from 4 hydrological stations, which are Wusheng station, Xiaoheba station, Luoduxi station and Beibei station. Data cover the period from 1961 to 2010.

The Jialing River basin is divided into three sub basins for detailed analysis, which are the Mainstream basin, the Qu River basin and the Fu River basin. Wusheng station, Xiaoheba station, Luoduxi station and Beibei station are the controlling hydrological stations of the Mainstream basin, the Fu River basin, the Qu River basin and the whole Jialing River basin respectively. The SPEI of each sub basin is calculated by applying the arithmetic averages of the monthly climate data at each meteorological station in each sub basin.

The spatial distribution of meteorological stations and sub basin division over the Jialing River basin is shown in S1 Fig. S1 Table gives the relationship of meteorological stations, controlling hydrological stations and sub basins.

## Methodology

### SPEI

For calculating the SPEI, the algorithm developed by Vicente-Serrano et al [14] is used. The documentation and executable files are freely available at http://digital.csic.es/handle/10261/10002. The computation of the SPEI is briefly introduced as follows. The detailed introduction of the SPEI can be found in [14].

(1) The calculation of potential evapotranspiration (PET). To calculate SPEI index, potential evapotranspiration (PET) applies the method described by Thornthwaite [23]. This represents a simple climatic water balance that is calculated at different time scales.

(2) The measurement of the water surplus or deficit of a climate water balance. With a value for PET, the difference between the precipitation *P* and PET for the month *i* is calculated according to:

$$D_{i}=P_{i}-{\text{PET}}_{i}$$ (1)

The probability distribution of cumulative *D*_*i*_ series is aggregated at different time scales, following the same procedure as that for the SPI.

(3) The normalization of the water balance. As there are many negative values in *D* series, the log-logistic distribution is selected for standardizing the *D* series. The probability density function of a three-parameter log-logistic distributed variable is expressed as:

$$f(x)=\frac{\beta }{\alpha }(\frac{x-\gamma }{\alpha }){\left[1+(\frac{x-\gamma }{\alpha })\right]}^{-2}$$ (2)

where *α*, *β* and *γ* are scale, shape, and origin parameters respectively, for *D* values in the range (*γ*>*D*<∞). The three parameters of the Pearson III distribution can be obtained as the following by Singh et al [24].

According to the log-logistic distribution, the probability distribution function for standardizing the *D* series for all time scales is given by:

$$F(x)={\left[1+{\left(\frac{\alpha }{x-\gamma }\right)}^{\beta }\right]}^{-1}$$ (3)

with *F*(*x*) the SPEI can easily be obtained as the standardized values of *F*(*x*). Following the classical approximation:

$$\text{SPEI}=W-\frac{C_{0}+C_{1}W+C_{2}W^{2}}{1+d_{1}W+d_{2}W^{2}+d_{3}W^{3}}$$ (4)

where in the above equation

$$W=\sqrt{-2\;\text{ln}(P)}\;\text{for}\;P\leq 0.5$$ (5)

*P* is the probability of exceeding a determined *D* value, *P* = 1−*F*(*x*). If P>0.5, then *P* is replaced by 1−*P* and the sign of the resultant SPEI is reversed. The constants are *C*_0_ = 2.515517, *C*_1_ = 0.802853, *C*_2_ = 0.010328, *d*_1_ = 1.432788, *d*_2_ = 0.189269, and *d*_3_ = 0.001308. The average value of the SPEI is 0, and the standard deviation is 1. The SPEI is a standardized variable, and it can therefore be compared with other SPEI values over time and space.

The SPEI is calculated on time scales of 3, 6, 9 and 12 months for the observations. Additionally, a moving-window is actually used to calculate the SPEI, which is used to analyze the change tendency of monthly water discrepancy between precipitation and potential evapotranspiration.

### SDI

Based on the SPI developing concepts, the SDI was developed by Nalbantis and Tsakiris [17] for characterizing hydrological drought at different time scales. In the Jialing River basin, hydrological year is from October to September of every next year, and so four overlapping time periods (hereafter as reference periods) are utilized within each hydrological year, which include October–December, October–March, October–June, and October–September (one complete hydrological year). For every hydrological year, the four overlapping time periods are always defined as October–December, October–March, October–June, and October–September respectively.

For the SPEI and SDI, the drought indices at different time scales represent the state of droughts having different durations in a basin. For example, the drought index for 3 months is used for reflecting the seasonal water status, which helps to support useful information for irrigation in agricultural production. The drought index for 6 months can reflect the drought condition in half a year. The drought index for 12 months can consider the impact of climatic change on regional water resources. Generally, the indices can stand for the dry and wet conditions of water conditions on a larger scale when the time scale getting longer.

A brief introduction of the SDI is presented as follows.

It is assumed that a time series of monthly streamflow volumes *Q*_*i*,*j*_ is available where *i* denotes the hydrological year and *j* is the month within that hydrological year, i.e. *j* = 1 for October and *j* = 12 for September. Based on this series, cumulative streamflow volume is computed as follows:

$$V_{i,k}=\sum_{j=1}^{3k}Q_{i,j}\;i=1,2,\ldots \;j=1,2,\ldots 12\;k=1,2,3,4$$ (6)

where *V*_i,k_ is the cumulative streamflow volume for the *i*-th hydrological year and the *k*-th reference period, *k* = 1 for October–December, *k* = 2 for October–March, *k* = 3 for October–June, and *k* = 4 for October–September.

The SDI is defined based on cumulative streamflow volumes *V*_i,k_ for each reference period *k* of the *i*-th hydrological year as follows:

$${\text{SDI}}_{i,k}=\frac{V_{i,k}-{\bar{V}}_{k}}{s_{k}}\;i=1,2,\ldots \;k=1,2,3,4$$ (7)

where $\bar{V_{k}}$ and *s*_*k*_ are respectively the mean and the standard deviation of cumulative streamflow volumes of reference period *k* as these are estimated over a long period of time. In this definition the truncation level is set to $\bar{V_{k}}$ although other values could be used.

In this work we use the two-parameter log-normal distribution for which the normalization is simple. It suffices taking natural logarithms of streamflow. The SDI index is then defined as:

$${\text{SDI}}_{i,k}=\frac{y_{i,k}-{\bar{y}}_{k}}{s_{y,}{}_{k}}\;i=1,2,\ldots \;k=1,2,3,4$$ (8)

in which

$$y_{i,k}=\text{ln}(V_{i,k})\;i=1,2,\ldots \;k=1,2,3,4$$ (9)

are the natural logarithms of cumulative streamflow with mean ${\bar{y}}_{k}$ and standard deviation *s*_*y*,*k*_ as these statistics are estimated over a long period of time. Positive SDI values reflect wet conditions while negative values indicate a hydrological drought. States of hydrological drought are defined by the SDI in an identical way to those used in the meteorological drought indices such as the SPI. S2 Table presents the grade categorization of climatic and hydrologic droughts defined by the SPEI and SDI [17].

### Mann-Kendall trend test

The rank-based non-parametric Mann–Kendall (MK) trend test [25], recommended by the World Meteorological Organization [26], is used here to detect the change in trends of the SPEI and SDI. The advantages of this method include (1) the ability to handle non-normality, censoring, or data reported as values ‘‘less than”, missing values, or seasonally and (2) having a high asymptotic efficiency [27–28]. The procedure of MK trend test performed in the study can be found in [25].

In this paper, two specific significance levels α = 0.05 and α = 0.01 (corresponding threshold values of the M-K value are ±1.96 and ±2.58 respectively) are used. When the M-K value is greater than 1.96 or less than -1.96, the change trend is significant. When the M-K value is greater than 2.58 or less than -2.58, the change trend is extremely significant.

### Correlation analysis

To analyze the relationship between climatic and hydrological droughts in sub basins and the whole basin, the Pearson correlation coefficients between the SDI and SPEI were calculated for different time scales. The Pearson coefficients are very suitable for analyzing the potential relationships between two independent variables [19, 29].

For instance, to analyze the relationships between the SDI and SPEI at the time scale of 3 months in the Mainstream basin, the SDI for October–December at Wusheng station, and the areal SPEI for October–December in the Mainstream basin are used to calculate the correlation coefficients.

To find the effects of previous climate conditions on hydrology, the SPEI having different lag times in sub basins are also used to calculate the correlation coefficients between the SPEI and SDI. The runoff of the Jialing river basin in one year is mainly concentrated from July to September, and the rainfall in one year is concentrated from May to September [30], which tends to show that the precipitation has one or two months lag impact on the hydrological process in the Jialing River basin. Therefore, the lag time for calculation was limited to 4 months. For the same example, referring to calculation of the correlation coefficients between the SDI and SPEI at the time scale of 3 months in the Mainstream basin, the areal SPEI calculated for October-December, for September-November, for August-October, for July-September, and for June-August in the Mainstream basin are used, to calculate the Pearson correlation coefficients between the SPEI and SDI for October–December at Wusheng station. After calculation, 5 correlation coefficients between the SDI and SPEI at different lag times are worked out. It is the same process for the other basins and the other time scales.

## Results and Discussions

### Temporal and spatial changes of climatic drought in the whole basin

#### Temporal changes

To analyze the temporal changes of climatic drought in the entire Jialing River basin, the SPEI of the 16 rain gauging stations are calculated for time scales of 3, 6, 9 and 12 months at first. Subsequently, the MK test is used to detect the trend of the SPEI.

S3 Table presents the MK values of the SPEI of the 16 rain gauging stations within the Jialing River basin or surrounding area for different time scales from 1962 to 2010. It can be seen that most of the stations are characterized by negative M-K values for different time scales. Furthermore most of the stations pass significant test at 95% confidence level, some of them even pass significant test at 99% confidence level. The results show that the increasing tendency of climatic drought dominates major parts of the Jialing River basin, and the increasing tendency in some stations is significant or extremely significant.

Climatic drought has the most obvious increasing trend at Minxian, Wudu, Songpan, Pingwu, Mianyang, Lueyang, Liangping and Guangyuan stations. The increasing tendencies in these 8 stations pass significant test at 99% confidence level for all time scales, which indicates that increasing trend of climatic drought at the 8 stations is extremely significant. The Liangping station is outside of the Jialing River basin, but the other 7 stations are all located in the northwest of the basin where the annual precipitation is less than it in the other parts of the basin. Du et al (2015) also found that the precipitation largely reduced in the northwest of the Jialing River basin recently. The reduction of precipitation is possible caused by the weakening of the Asian monsoon circulation [31]. More notable is that the northwest of the basin has a complicated terrain and geology. The ecosystem in this area is very vulnerable to human activities, which can be another reason for the reduction of precipitation and the increasing trend of climatic drought.

Daxian station is the only station whose M-K values for all time scales are positive, which means that climatic drought has a weak decreasing tendency in Daxian station. However, none of M-K values for this station is significant at 95% confidence level. Wanyuan station has the similar situation like Daxian. The reason might be that both of the two stations are located in the east of the Qu River basin. The southeast of the Qu River basin is largely effected by the Asian monsoon circulation and has a mountainous terrain, which can together amplify the precipitation effect in this area.

#### Spatial changes

In order to analyze the spatial changes of climatic drought, the MK values of the 16 stations for time scales of 3, 6, 9 and 12 months are interpolated respectively by the inverse distance weighting (IDW) method.

S2 Fig shows the spatial changes of the climatic drought representing by MK values for time scales of 3, 6, 9 and 12 months. It can be seen that most of the basin are colored in dark grey for time scales of 3, 6, 9 and 12 months, which shows most of the Jialing River basin experience extremely significant increasing drought tendency for all time scales.

Additionally, the Jialing River basin shows obvious regional differences in spatial distribution. Climatic drought has an increasing trend in most of the basin, especially in the western and northern basin. Climatic drought doesn’t have obvious increasing tendency in eastern basin, and several stations in eastern basin seven how a slight decreasing tendency. The spatial variations of the climatic drought are consistent with the spatial distribution of the annual precipitation and terrain in the basin. The southeast of the basin is affected by the Asian monsoon, and the precipitation is abundant, while the northwest region is weakly affected by the Asian monsoon, and the precipitation is relatively small. Secondly, altitude is also an important factor affecting the precipitation. The altitude of the northwest region is higher in the basin, which causes the less precipitation. Since the precipitation is a dominant factor for calculating the SPEI, the spatial variations of climatic drought have the very similar pattern as the annual precipitation in the Jialing River basin. As time scales grow from 3 months to 12 months, more area in the Jialing River basin experience significant increasing tendency for climatic drought, which suggests that the increasing tendency of climatic drought is more obvious for longer time scales in the Jialing River basin.

### Temporal changes of climatic and hydrological droughts in sub basins

To assess the changes of climatic and hydrological droughts in different basins, the averaged SPEI of all rain gauge stations in the sub basins and the SDI of controlling hydrological stations are calculated for different time scales. Besides the whole Jialing River basin, the three other basins are the Mainstream basin, the Fu River basin, and the Qu River basin.

#### The Mainstream basin

As can be seen from S1 Table, 6 rain gauging stations are averaged for the Jialing mainstream basin and Wusheng hydrological station is used as controlling hydrological station for this basin. S3 Fig shows the SPEI and SDI for time scales of 3, 6, 9 and 12 months during 1962–2010. It can be seen that the SPEI and SDI are consistent with each other at most of the study period. For all time scales, the SPEI and SDI show decreasing tendency, which indicates that the climatic and hydrological droughts tend to increase from 1962 to 2010 in the mainstream basin.

The trend of the SPEI and SDI is detected by MK test (S4 Table). The Mainstream basin is characterized by negative M-K values for all time scales. Except that the SDI for time scale of 6 months passes significant test at 95% confidence level, others pass significant test at 99% confidence level.

Obviously, the Mainstream basin experiences the significant increasing trends for climatic and hydrological droughts. The increasing trend of climatic drought is more significant than hydrological drought. Moreover, the increasing trend of climatic drought is more obvious for longer time scales, so does hydrological drought.

#### The Fu River basin

According to S1 Table, 4 rain gauge stations are averaged for the Fu River basin and Xiaoheba hydrological station is chosen as the controlling hydrological station. S4 Fig presents the SPEI and SDI during 1962–2010 for time scales of 3, 6, 9 and 12 months in Fu River basin. Similar with the Mainstream basin, the SPEI and SDI are consistent with each other at most of the study period. In addition, the SPEI and SDI tend to decrease obviously on longer time scales, especially for 9 and 12 months.

S5 Table presents MK value of the SPEI and SDI for different time scale. The Fu River basin is characterized by negative M-K values which indicate that Fu River basin has an increasing trend for droughts.

However, the increasing trends of climatic drought and hydrological drought have some differences. The SPEI for all time scales pass significant test at 99% confidence level. The MK value of the SPEI becomes smaller and smaller with the increase of time scale, which shows that the increasing trend of climatic drought is more significant for longer time scales. The increasing trend of hydrological drought is not that much significant. Only the SDI for time scale of 9 and 12 months pass significant test at 95% confidence level.

#### The Qu River basin

For the Qu River basin, 4 rain gauge stations are averaged to compute the areal SPEI and Luoxidu hydrological station is chosen as the controlling hydrological station according to S1 Table. S5 Fig presents the SPEI and SDI during 1962–2010 for time scales of 3, 6, 9 and 12 months in the Qu River basin. Similar with the other sub basins, the SPEI and SDI of the Qu River basin are consistent with each other at most of the study period. Unlike the other two sub basins, the decreasing trends of climatic and hydrological droughts in the Qu River basin are less significant.

S6 Table presented MK value of the SPEI and SDI for different time scales. The Qu River basin is characterized by negative M-K values which indicate that climatic and hydrological droughts in the Qu River basin have an increasing tendency.

However the increasing tendency in the Qu River basin is inconspicuous when comparing to that of the Mainstream basin and the Fu River basin. None of the SDI passes significant test at 95% confidence level, which means that the increasing tendency of hydrological drought is not significant in the Qu River basin. The MK value of the SPEI becomes smaller and smaller with the increase of time scale, which shows that the increasing trend of climatic drought is more significant for longer time scales. The SPEI on time scale of 6, 9 and 12 months passes significant test at 95% confidence level.

#### The whole Jialing River basin

All of the 16 rain gauge stations are averaged for the whole Jialing River basin and Beibei hydrological station is the controlling hydrological station. S6 Fig presents the SPEI and SDI for time scales of 3, 6, 9 and 12 months during 1962–2010 in the Jialing River basin. For short time scales including 3 and 6 months, the changes of the SPEI and SDI are very similar. For longer time scales including 9 and 12 months, the variation ranges of the SPEI and SDI are smaller than them under shorter time scales.

S7 Table presents MK value of the SPEI and SDI for different time scales. The whole Jialing River basin is characterized by negative M-K values which indicate that the whole basin has an increasing trend for droughts. Like all the sub basins, the increasing trend of climatic drought is more significant than that of hydrological drought. All of the SPEI pass significant test at 99% confidence level, which means that the increasing trend of climatic drought is extremely significant in the whole Jialing River basin, especially for longer time scales such as 12 months. For the SDI, only it on time scale of 12 months passes significant test at 95% confidence level.

Generally, the climatic and hydrological droughts in the whole Jialing River basin and sub basins show the increasing tendency, which possible relates with the weakening of the Asian monsoon. It was found that the climatic droughts in the Jialing River basin tended to increase recently [32–33]. In addition, the increasing tendency of climatic and hydrological droughts is more obvious with the increase of time scale, and the droughts on longer time scales are mainly determined by rainfall. The hydrological characteristics of the Jialing River basin are having obvious dry season and rainy season. Rainfall in the main flood season (from June to September) is the most abundant in one year and can count 66% of annual precipitation. Research [30] showed that annual precipitation had decreasing tendency and seasonal rainfall was relatively concentrated. Therefore, the SPEI on longer time scales has bigger variations than it on shorter time scales due to the gradual changes of precipitation factor.

It should also be noted that the changes of climate and hydrological droughts in the sub basins of the Jialing River basin are not quite the same. The Mainstream basin and the Fujiang River basin have the similar tendencies. The two sub basins locate in the western and northern parts of the basin, where annual precipitation is smaller than other parts. Partly, the similar variations of the SPEI in the two sub basins reflect that the temperature has been increased obviously in the upper and middle reaches of the Jialing River basin, which could have impacts on potential evapotranspiration and water discrepancy. The climatic and hydrological droughts in the Qu River basin are not significant comparing to the other two sub basin, partly because the Qu River basin belongs to subtropical monsoon area and has abundant precipitation.

### Relationship between climatic and hydrological droughts

To analyze the relationship between climatic and hydrological droughts in sub basins and the whole basin, the correlation coefficients between the SDI and SPEI at different lead times are calculated for different time scales (S8 Table).

For time scale of 3 months and 6 months, the relationships between climatic and hydrological droughts in different basins are different. The Mainstream basin and the Fu river basin have the similar situation. The correlation coefficient between the SDI and SPEI reaches the biggest when the start month of the SPEI is one month earlier than the SDI, secondly is two months earlier, thirdly is the same as the SDI, which shows that the time lag between the SDI and SPEI is around one or two months, especially for the Mainstream basin. The occurrence of climatic droughts is one or two month earlier than the hydrological droughts in these two basins. Hence, the occurrence of climatic drought two month earlier could be an early warning signal of hydrological drought, and the occurrence of climatic drought one month earlier usually could cause the occurrence of hydrological drought. For the Qu river basin, the correlation coefficient between the SDI and SPEI reaches the biggest when the start month of the SPEI is the same as the SDI, which shows that hydrological droughts and climatic droughts occur in the same time.

For the whole Jialing River basin, the correlation coefficient between the SDI and SPEI reaches the biggest when the start month of the SPEI is the same as the SDI and one month earlier than the SDI, which shows that hydrological droughts are affected by climatic droughts occurring one month before and also the simultaneous climatic droughts. Interestingly, the correlation coefficients for all the basins become smaller when the start month of the SPEI is two months earlier than the SDI, which means that the hydrological droughts are less affected by rainfall in three months ago.

For time scale of 9 months and 12 months, the results are very similar. In all the basins, the correlation coefficient between the SDI and SPEI reaches the biggest when the start month of the SPEI is the same as the SDI, and the coefficients under the time scale of 12 months are significantly larger than them under the time scale of 9 months. The results show that occurrences of hydrological droughts and climatic droughts on longer time scales (especially for annual time scale) are basically simultaneous.

Additionally, correlation coefficients for 12 months are greater than or equal to 0.7 when time lag is zero or one month, which are almost the biggest among the four different time scales. The higher values indicate that climate has a significant impact on hydrological process on annual time scale. But for shorter time scales, human activities would have obvious impacts on hydrology and water resources. In the upper reaches of the Jialing River basin, some reservoirs have been constructed. Normally, the impacts of hydraulic engineering occur within one hydrological year. The reservoirs could change the temporal distribution of hydrological processes in one hydrological year. They could increase the water quantity in the rivers in dry season (especially from late autumn to winter) and could decrease stream discharge in flood season, which cause the lower correlation coefficients between climatic droughts and hydrological droughts on shorter time scales.

## Conclusions

This paper investigates the changes of climatic and hydrological droughts in the Jialing River basin from 1962 to 2010, as represented by the SPEI and SDI calculated with various time scales (3, 6, 9 and 12 months). The characteristics of climatic and hydrological droughts are assessed both at the entire Jialing River basin and at the regional levels of the three sub basins. Our results provide a comprehensive assessment of climatic and hydrological droughts in terms of temporal and spatial evolution based on the newer drought indices SPEI and SDI. The main results are summarized as follows:

1. Climatic drought has an increasing trend in the Jialing River basin. The increasing trend of climatic drought is significant (passing the significant test at 95% confidence level) or extremely significant (passing the significant test at 99% confidence level) in most of the basin, while hydrological drought has a less significant increasing trend. For the spatial distribution, climatic drought has the significant increasing trend in the western and northern basin, while climatic drought doesn’t have obvious increasing trend in eastern basin, and several stations in eastern basin even show a very slight decreasing tendency.
2. The three sub basins in the Jialing River basin show different increasing trends for hydrological droughts. Hydrological drought has the most significant increasing trend in the Mainstream basin, while the increasing trend in the Fu River basin is not quite significant, and the increasing trend in the Qu River basin is the least significant. The differences of hydrological drought among the three sub basins are consistent with the spatial distribution of climatic drought.
3. For the time scale of 3 months and 6 months, the correlation coefficients for all the basins become smaller when the start month of the SPEI is one or two months (especially one month) earlier than the SDI, which means that hydrological droughts are less affected by rainfall in three months ago. Relationships between climatic and hydrological droughts in the mainstream basin and the Fu River basin are more consistent than the Qu River basin. However, the result gets different as time scales becoming longer. For the time scale of 9 months and 12 months, the correlation coefficients between the SDI and SPEI in all the basins reaches the biggest when the start month of the SPEI is the same as the SDI, which shows that occurrences of climatic and hydrological droughts on longer time scales are basically simultaneous in the Jialing River basin.
4. In addition, the correlation coefficients between climatic droughts and hydrological droughts are getting smaller when lag time getting longer. On shorter time scales (no longer than one hydrological year), human activities would have obvious impacts on hydrology and water resources due to the effects of the reservoirs on temporal distribution of stream discharge.

Generally, the SPEI and SDI are very suitable for assessing the variations of climatic and hydrological droughts and the two indices are applicable to analyze the relationships between climatic and hydrological droughts. Under the condition of global and regional climate change, the SPEI considering the effects of temperature can test out the changes of climatic drought in more effective way.

Based on the assessment, hydrological droughts at different time scales have different connections with climatic droughts for sub basins of the Jialing River basin. On short time scales, it seems that climatic droughts have one or two months lag impact on hydrological droughts in the north-west area of the basin, and has one month lag impact in south-east area of the basin, which can be the useful information for predicting hydrological droughts and alleviating the negative impacts. Additionally, the droughts (including climatic and hydrological droughts) increased during the recent several decades in the Jialing River basin, and they would still tend to increase in the next few years, which should have influences on water resources management and social development in the basin.

## Supporting Information

S1 Dataset The original monthly climate data at some stations. (XLSX)

S2 Dataset The metadata used for the results in the manuscript. (XLSX)

S1 Fig Locations of meteorological stations and hydrological stations in the Jialing River basin. (DOCX)

S2 Fig Spatial changes of SPEI at different time scales in the Jialing River basin (A)3 months (B) 6 months (C) 9 months (D) 12 months. (DOCX)

S3 Fig Variations of the SPEI and SDI for time scales of 3, 6, 9 and 12 months during 1962–2010 for the Mainstream basin (a) SPEI-3 (b) SDI-3 (c) SPEI-6 (d) SDI-6 (e) SPEI-9 (f) SDI-9 (g) SPEI-12 (h) SDI-12. (DOCX)

S4 Fig Variations of the SPEI and SDI for time scales of 3, 6, 9 and 12 months during 1962–2010 for the Fu River basin (a) SPEI-3 (b) SDI-3 (c) SPEI-6 (d) SDI-6 (e) SPEI-9 (f) SDI-9 (g) SPEI-12 (h) SDI-12. (DOCX)

S5 Fig Variations of the SPEI and SDI for time scales of 3, 6, 9 and 12 months during 1962–2010 for the Qu River basin (a) SPEI-3 (b) SDI-3 (c) SPEI-6 (d) SDI-6 (e) SPEI-9 (f) SDI-9 (g) SPEI-12 (h) SDI-12. (DOCX)

S6 Fig Variations of the SPEI and SDI for time scales of 3, 6, 9 and 12 months during 1962–2010 for the Jialing River basin (a) SPEI-3 (b) SDI-3 (c) SPEI-6 (d) SDI-6 (e) SPEI-9 (f) SDI-9 (g) SPEI-12 (h) SDI-12. (DOCX)

S1 Table Meteorological stations and hydrological stations in sub basins. (DOCX)

S2 Table Classification used for the SPEI and SDI according to their values. (DOCX)

S3 Table M-K values of the SPEI during 1962–2010 for different time scales. (DOCX)

S4 Table M-K values of the SPEI and SDI for the Mainstream basin. (DOCX)

S5 Table M-K values of the SPEI and SDI for the Fu River basin. (DOCX)

S6 Table M-K values of the SPEI and SDI for the Qu River basin. (DOCX)

S7 Table M-K values of the SPEI and SDI for the Jialing River basin. (DOCX)

S8 Table Correlation coefficients between the SDI and SPEI for different time scales. (DOCX)
