# Supplementary figures and images for: Changes and Relationships of Climatic and Hydrological Droughts in the Jialing River Basin, China
Source: PLoS One. 2015 Nov 6;10(11):e0141648. doi: 10.1371/journal.pone.0141648 (PMC4636145; doi:10.1371/journal.pone.0141648)

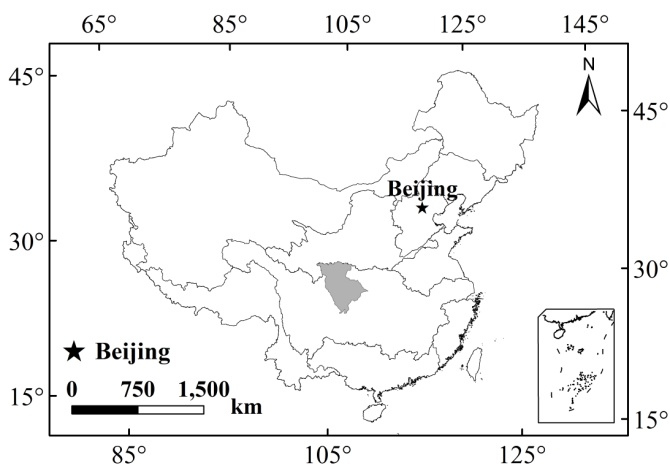


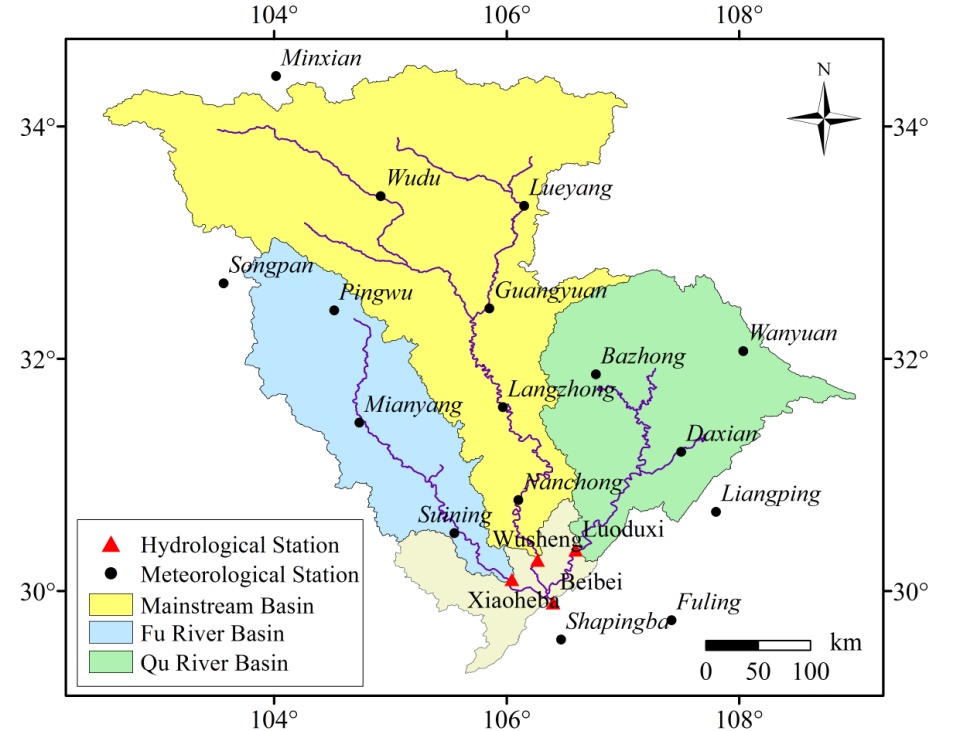

Supplement: S1 Fig — (DOCX) [file pone.0141648.s003.docx]

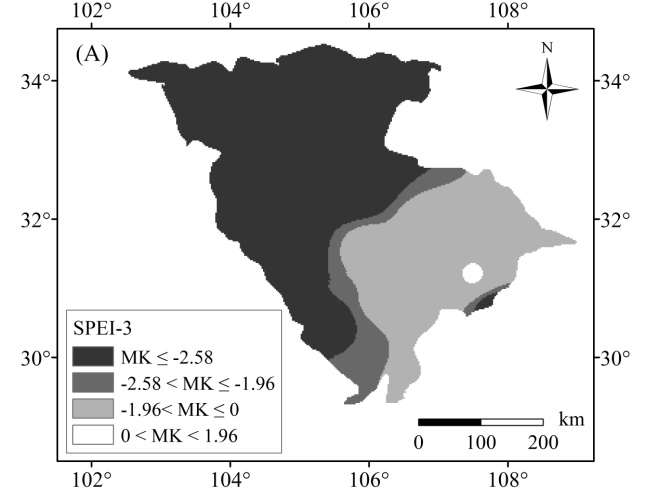

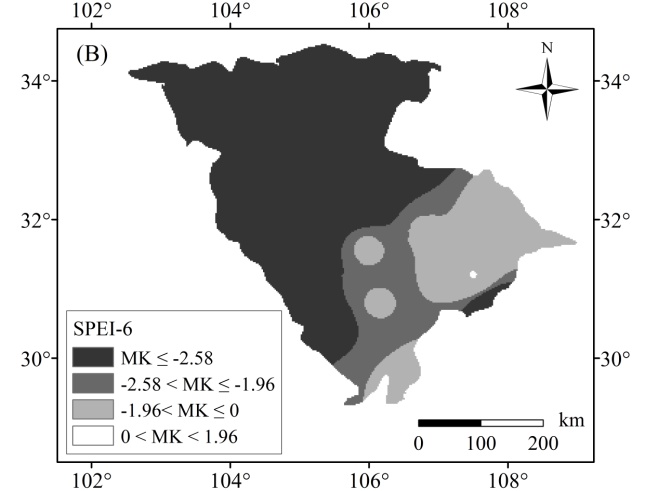


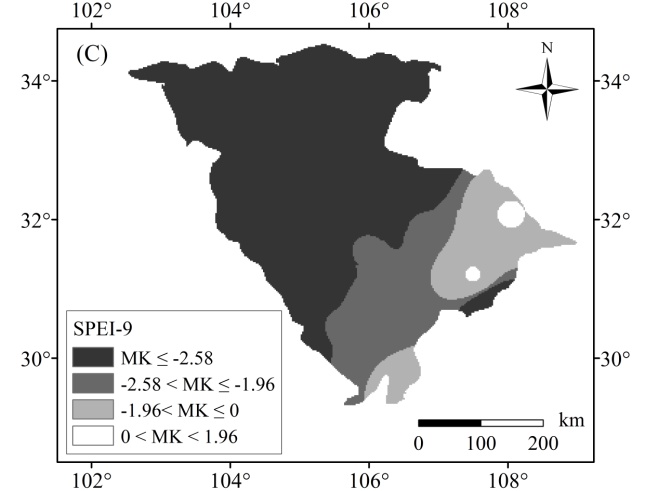

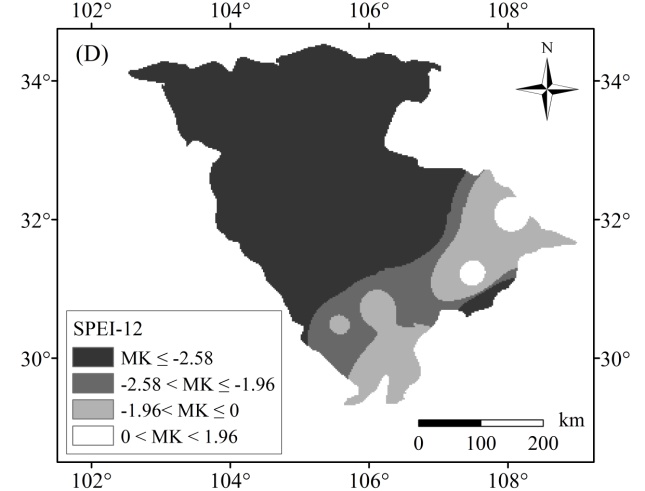

Supplement: S2 Fig — (DOCX) [file pone.0141648.s004.docx]
