# Supplementary material for: Changes and Relationships of Climatic and Hydrological Droughts in the Jialing River Basin, China
Source: PLoS One. 2015 Nov 6;10(11):e0141648. doi: 10.1371/journal.pone.0141648 (PMC4636145; doi:10.1371/journal.pone.0141648)
Supplement: S1 Table — (DOCX) [file pone.0141648.s009.docx]

| Basin | Hydrological Station | Meteorological station |
| --- | --- | --- |
| the Mainstream basin | Wusheng | Minxian, Wudu, Lueyang, Guangyuan, Langzhong, Nanchong |
| the Fu River basin | Xiaoheba | Songpan, Pingwu, Mianyang, Suining |
| the Qu River basin | Luoduxi | Wanyuan, Bazhong, Daxian, Liangping |
| the Jialing River basin | Beibei | All 16 rain gauge stations |
