# Supplementary material for: Changes and Relationships of Climatic and Hydrological Droughts in the Jialing River Basin, China
Source: PLoS One. 2015 Nov 6;10(11):e0141648. doi: 10.1371/journal.pone.0141648 (PMC4636145; doi:10.1371/journal.pone.0141648)
Supplement: S2 Table — (DOCX) [file pone.0141648.s010.docx]

| Categories of the SPEI and SDI | SPEI & SDI |
| --- | --- |
| Non-drought | <0.0 |
| Mild drought | -1 to 0 |
| Moderate drought | −1.5 to -1.01 |
| Severe drought | −2 to -1.51 |
| Extreme drought | <-2 |
