# Supplementary material for: Changes and Relationships of Climatic and Hydrological Droughts in the Jialing River Basin, China
Source: PLoS One. 2015 Nov 6;10(11):e0141648. doi: 10.1371/journal.pone.0141648 (PMC4636145; doi:10.1371/journal.pone.0141648)
Supplement: S3 Table — (DOCX) [file pone.0141648.s011.docx]

| Stations | 3 months | 6 months | 9 months | 12 months |
| --- | --- | --- | --- | --- |
| Minxian | -5.51^**^ | -8.28^**^ | -9.72^**^ | -10.71^**^ |
| Wudu | -5.93^**^ | -6.53^**^ | -7.00^**^ | -8.06^**^ |
| Songpan | -2.64^**^ | -4.21^**^ | -5.29^**^ | -6.31^**^ |
| Pingwu | -3.47^**^ | -5.28^**^ | -6.49^**^ | -7.86^**^ |
| Mianyang | -4.59^**^ | -4.91^**^ | -5.34^**^ | -6.40^**^ |
| Lueyang | -4.51^**^ | -4.46^**^ | -4.63^**^ | -5.17^**^ |
| Guangyuan | -4.68^**^ | -5.23^**^ | -6.18^**^ | -7.23^**^ |
| Wanyuan | -1.05 | -0.30 | 0.32 | 0.55 |
| Langzhong | -0.67 | -1.58 | -2.18^*^ | -2.65^**^ |
| Bazhong | -0.66 | -1.78 | -2.39^*^ | -3.05^**^ |
| Daxian | 0.36 | 0.06 | 0.28 | 0.88 |
| Suining | -3.15^**^ | -3.04^**^ | -2.52^*^ | -1.87 |
| Nanchong | -1.62 | -1.77 | -1.97^*^ | -1.69 |
| Liangping | -3.86^**^ | -5.35^**^ | -6.05^**^ | -6.89^**^ |
| Shapingba | -1.92 | -1.50 | -1.50 | -0.84 |
| Fuling | -0.68 | -0.61 | -0.21 | 0.59 |

^*^ means passing significant test at 95% confidence level.

^**^ means passing significant test at 99% confidence level.

It is the same for S4 to S7 Tables.
