# Supplementary material for: Changes and Relationships of Climatic and Hydrological Droughts in the Jialing River Basin, China
Source: PLoS One. 2015 Nov 6;10(11):e0141648. doi: 10.1371/journal.pone.0141648 (PMC4636145; doi:10.1371/journal.pone.0141648)
Supplement: S4 Table — (DOCX) [file pone.0141648.s012.docx]

| Index | M-K value for 3 months | M-K value for 6 months | M-K value for 9 months | M-K value for 12 months |
| --- | --- | --- | --- | --- |
| SPEI | -5.12^**^ | -6.1^**^ | -7.01^**^ | -7.80^**^ |
| SDI | -2.63^**^ | -2.40^*^ | -2.90^**^ | -2.65^**^ |
