# Supplementary material for: Changes and Relationships of Climatic and Hydrological Droughts in the Jialing River Basin, China
Source: PLoS One. 2015 Nov 6;10(11):e0141648. doi: 10.1371/journal.pone.0141648 (PMC4636145; doi:10.1371/journal.pone.0141648)
Supplement: S5 Table — (DOCX) [file pone.0141648.s013.docx]

| Index | M-K value for 3 months | M-K value for 6 months | M-K value for 9 months | M-K value for 12 months |
| --- | --- | --- | --- | --- |
| SPEI | -4.71^**^ | -5.83^**^ | -6.80^**^ | -7.83^**^ |
| SDI | -1.10 | -0.23 | -2.27^*^ | -2.20^*^ |
