# Supplementary material for: Changes and Relationships of Climatic and Hydrological Droughts in the Jialing River Basin, China
Source: PLoS One. 2015 Nov 6;10(11):e0141648. doi: 10.1371/journal.pone.0141648 (PMC4636145; doi:10.1371/journal.pone.0141648)
Supplement: S6 Table — (DOCX) [file pone.0141648.s014.docx]

| Index | M-K value for 3 months | M-K value for 6 months | M-K value for 9 months | M-K value for 12 months |
| --- | --- | --- | --- | --- |
| SPEI | -1.23 | -1.97^*^ | -2.01^*^ | -2.27^*^ |
| SDI | -1.03 | -1.34 | -0.25 | -1.71 |
