# Supplementary material for: Changes and Relationships of Climatic and Hydrological Droughts in the Jialing River Basin, China
Source: PLoS One. 2015 Nov 6;10(11):e0141648. doi: 10.1371/journal.pone.0141648 (PMC4636145; doi:10.1371/journal.pone.0141648)
Supplement: S8 Table — (DOCX) [file pone.0141648.s016.docx]

| Time scale | Basin | *t* | *t*-1 | *t*-2 | *t*-3 | *t*-4 |
| --- | --- | --- | --- | --- | --- | --- |
| 3 months | the Mainstream basin | 0.63 | 0.78 | 0.70 | 0.56 | 0.35 |
|  | the Fu River basin | 0.46 | 0.64 | 0.57 | 0.42 | 0.28 |
|  | the Qu River basin | 0.68 | 0.55 | 0.38 | 0.22 | 0.11 |
|  | the whole Jialing River basin | 0.70 | 0.70 | 0.50 | 0.32 | 0.17 |
| 6 months | the Mainstream basin | 0.62 | 0.74 | 0.71 | 0.67 | 0.68 |
|  | the Fu River basin | 0.53 | 0.57 | 0.52 | 0.44 | 0.45 |
|  | the Qu River basin | 0.60 | 0.44 | 0.38 | 0.27 | 0.27 |
|  | the whole Jialing River basin | 0.67 | 0.63 | 0.56 | 0.48 | 0.47 |
| 9 months | the Mainstream basin | 0.62 | 0.49 | 0.35 | 0.41 | 0.35 |
|  | the Fu River basin | 0.56 | 0.43 | 0.28 | 0.26 | 0.28 |
|  | the Qu River basin | 0.55 | 0.25 | 0.04 | -0.15 | -0.23 |
|  | the whole Jialing River basin | 0.60 | 0.43 | 0.27 | 0.17 | 0.11 |
| 12 months | the Mainstream basin | 0.91 | 0.83 | 0.64 | 0.46 | 0.44 |
|  | the Fu River basin | 0.81 | 0.70 | 0.42 | 0.14 | 0.24 |
|  | the Qu River basin | 0.89 | 0.77 | 0.53 | 0.18 | 0.02 |
|  | the whole Jialing River basin | 0.90 | 0.82 | 0.62 | 0.39 | 0.35 |

Note: *t* means that the SDI and SPEI have the same time period, *t*-1 means that start month and end month of the SPEI are one month earlier than the SDI, so do *t*-2, *t*-3 and *t*-4.
