# Supplementary material for: Changes and Relationships of Climatic and Hydrological Droughts in the Jialing River Basin, China
Source: PLoS One. 2015 Nov 6;10(11):e0141648. doi: 10.1371/journal.pone.0141648 (PMC4636145; doi:10.1371/journal.pone.0141648)

(a) SPEI-3

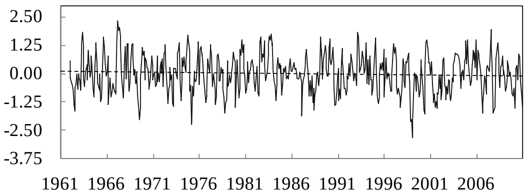

(b) SDI-3

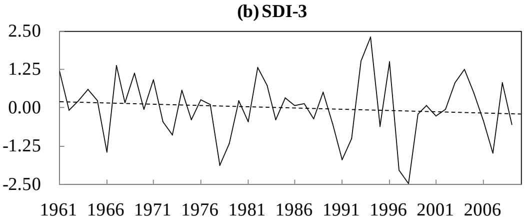

(c) SPEI-6

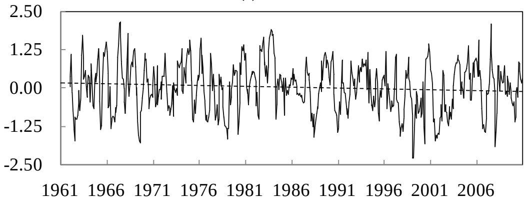

(d) SDI-6

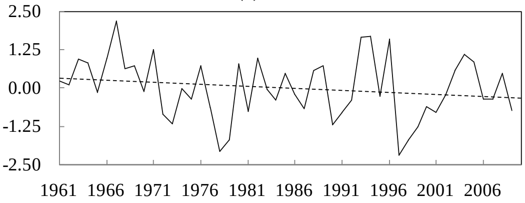

(e) SPEI-9

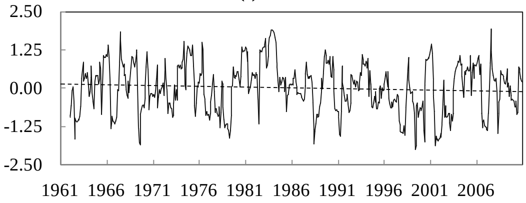

(f) SDI-9

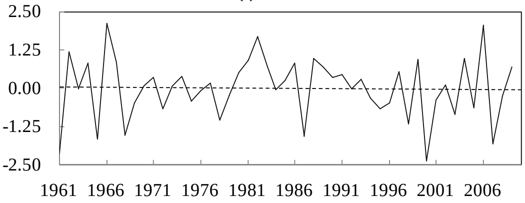

(g) SPEI-12

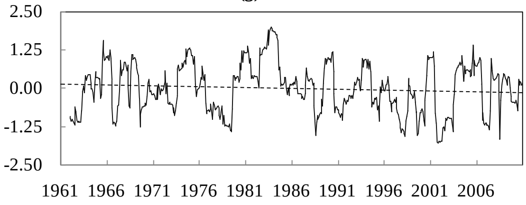

(h) SDI-12

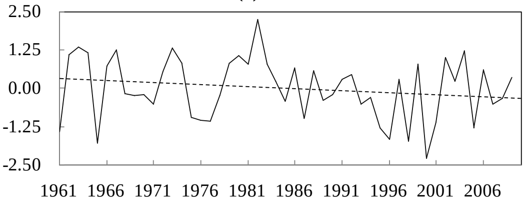

Supplement: Supplementary file 14 [file pone.0141648.s007.docx]
