# Supplementary material for: Changes and Relationships of Climatic and Hydrological Droughts in the Jialing River Basin, China
Source: PLoS One. 2015 Nov 6;10(11):e0141648. doi: 10.1371/journal.pone.0141648 (PMC4636145; doi:10.1371/journal.pone.0141648)
Supplement: Supplementary file 15 [file pone.0141648.s008.docx]

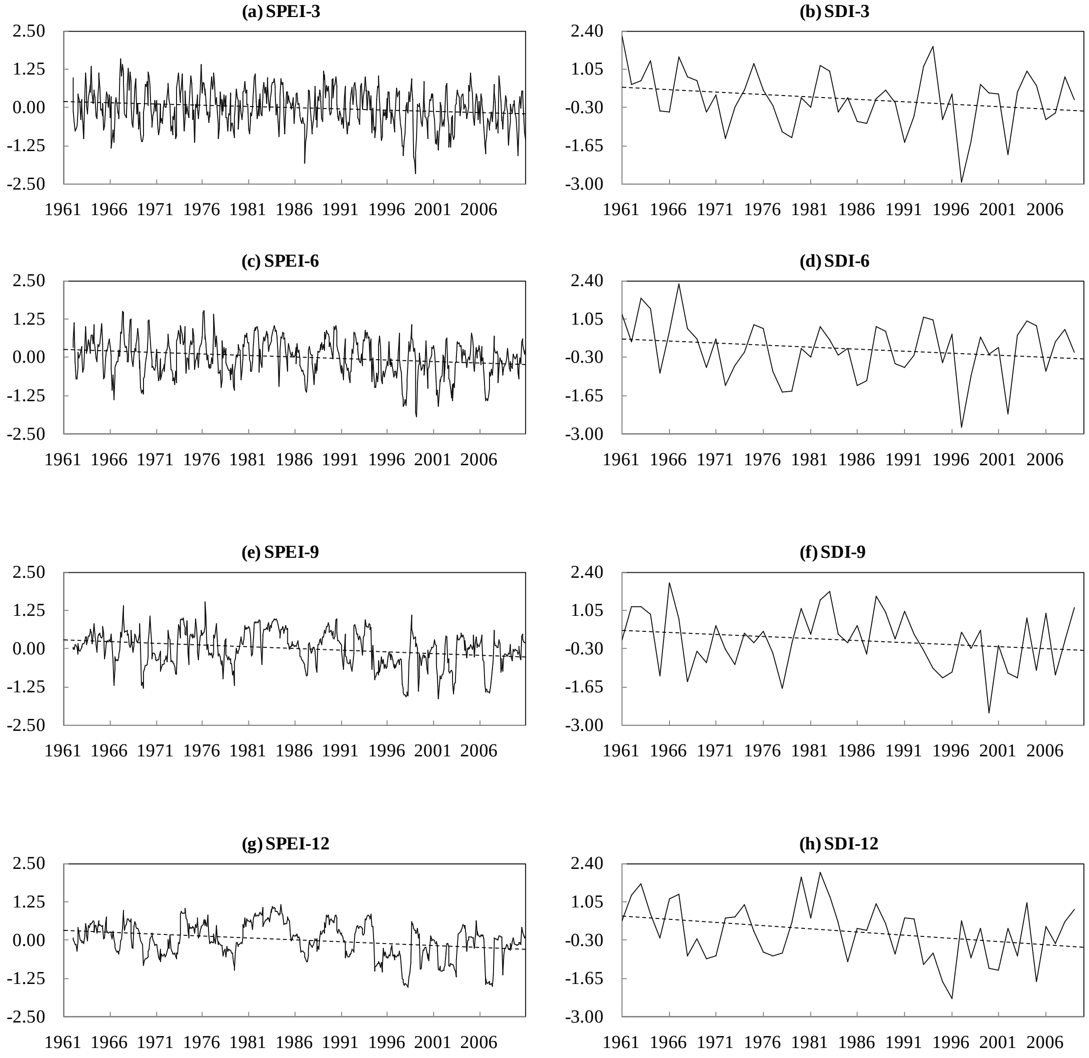
(a) SPEI-3
(b) SDI-3
(c) SPEI-6
(d) SDI-6
(e) SPEI-9
(f) SDI-9
(g) SPEI-12
(h) SDI-12
2.50
1.25
0.00
-1.25
-2.50
2.40
1.05
-0.30
-1.65
-3.00
1961 1966 1971 1976 1981 1986 1991 1996 2001 2006
